# Exploring the interplay of mindfulness, self-efficacy, and burnout among Chinese preschool teachers: a network approach

**DOI:** 10.3389/fpsyg.2025.1483099

**Published:** 2025-03-12

**Authors:** Lingyan Yan, Yunxiang Lin, Wenjie Li, Changsheng Hu

**Affiliations:** ^1^School of Communication, Fujian Normal University, Fuzhou, Fujian, China; ^2^The Research Center for Marxism and Contemporary Media, Fujian Social Science Research Base, Fuzhou, Fujian, China; ^3^School of Physical Education and Sport Science, Fujian Normal University, Fuzhou, Fujian, China; ^4^School of Psychology, Fujian Normal University, Fuzhou, Fujian, China; ^5^School of Teacher Education, Sichuan Vocational and Technical College, Suining, Sichuan, China

**Keywords:** preschool teachers, teaching mindfulness, self-efficacy, teacher burnout, network analysis

## Abstract

**Background:**

Teacher burnout is associated with a series of negative outcomes for teachers and children. Previous studies have confirmed the impact of teaching mindfulness and teacher self-efficacy on teacher burnout, but the relationship between them needs further research.

**Methods:**

This study was conducted in May 2024 on 572 kindergarten teachers in Sichuan and Yunnan provinces using the *Mindfulness in Teaching Scale*, the *Teacher Sense of Self-Efficacy Scale*, the *Teacher Burnout Scale*, and SPSS software for descriptive statistical analysis, and R software for network analysis.

**Results:**

The teacher mindfulness–teacher burnout network had 20 cross-community edges, the strongest of which were M1, “Non-automated instruction;” J2, “Depersonalization;” and M7, “Appropriate expression of pain,” which is demonstrated by these values displaying the highest bridge expected influences. The teacher self-efficacy-teacher burnout network had 16 cross-community edges, the strongest of which were S2, “Encouraging young children to love learning;” J3, “Low achievement;” and S11, “Home-school cooperation to help young children,” demonstrated by these values displaying the highest bridge expected influences.

**Conclusion:**

This study explored the relationships between teaching mindfulness, teacher self-efficacy, and teacher burnout using network analysis methods. The teaching mindfulness factor M7, “Appropriate expression of pain,” and the teacher self-efficacy factor S11, “Home-school cooperation to help young children,” showed negative correlations with various burnout factors. These factors were identified as the highest bridge centrality nodes, suggesting a stronger association with teacher burnout than other factors. Thus, they represent the optimal targets for interventions aimed at reducing teacher burnout.

## 1 Introduction

The *Standards for Teacher Professional Competence of Teacher Trainees in Preschool Education (Trial)*, issued by the Ministry of Education in 2021, states that preschool teachers should establish a spirit of love and dedication to their work, and be able to fulfill their responsibilities in educational practice conscientiously, actively study, be caring and responsible, and work with care and patience. China's *Code of Ethics for Teachers* also suggests that love and dedication are the essential requirements of the teaching profession. However, there are often news reports of kindergarten teachers abusing and beating young children (Li, 2021). This phenomenon may be linked to the fact that some groups of kindergarten teachers no longer have the initial enthusiasm and intention for their work and have become “burned out” by the teaching profession (Stapleton, 2024). Teacher burnout (job burnout, JB) is a state of physical, psychological, and behavioral exhaustion caused by high workload, long hours, and intense work demands (Li, 2003; Cherniss, 1980). It includes three dimensions: emotional exhaustion, depersonalization, and reduced personal accomplishment (Maslach et al., 2001). Kindergarten teachers are a high-risk group for burnout in China (Fan, 2019).

Previous studies have indicated a correlation between preschool teachers' burnout and their own physical and mental health (Li et al., 2020), as well as a potential negative association with young children's growth (Kim and Singh, 2018). For example, according to the person-environment fit theory, Mingming et al. (2024) indicated that when kindergarten teachers experience burnout, teacher turnover frequently occurs, which may be associated with impacts on the quality of education in kindergartens. Furthermore, a nationwide survey of rural kindergarten teachers in China conducted by Zhao et al. (2023) revealed a correlation between teacher burnout and teacher-child classroom conflicts, which also negatively influences childcare management. Finally, the research of He et al. (2024) found that long-term burnout negatively impacts the physical and mental health of kindergarten teachers, potentially impacting their work enthusiasm and efficiency, thus affecting teaching quality and interactions with children, and ultimately influencing children's emotional, behavioral, and cognitive development. Therefore, it is particularly important to explore the factors that influence preschool teacher burnout and carry out targeted measures to reduce the physical and mental health problems of preschool teachers and promote the development of young children.

Xanthopoulou et al.'s (2007) *Job Demands-Resources Model* proposes that mindfulness in teaching as a psychological work resource can maintain occupational health and effectively alleviate teacher burnout induced by social, organizational, or personal factors. Teaching mindfulness refers to a teacher's ability to consciously be aware of their internal, immediate experiences and external environmental stimuli with a curious, accepting, and non-judgmental attitude. It involves both personal and interpersonal mindfulness elements (Frank et al., 2016). The former is the teacher's ability to focus on the present moment without judgment, being aware of their feelings, thoughts, and actions during teaching. The latter refers to the teacher's ability to maintain openness, acceptance, and refrain from immediate reactions during teacher-student interactions (Barata-Gonçalves et al., 2024). Teaching mindfulness not only enables teachers to have a clear understanding of their own and students' states but also allows them to be aware of and accept the emotions and needs of both parties in the teacher-student interaction (Ding et al., 2020). Moreover, related studies have shown that teaching positive thinking is negatively related to teacher burnout. For example, Cheng et al. (2022) found that mindfulness in teaching was significantly negatively related to burnout and discussed it as a protective factor in reducing burnout among preschool teachers (Chen, 2020). In addition, Taylor et al.'s (2021) study also demonstrated that preschool teachers' levels of teaching mindfulness increased after a short-term mindfulness intervention and burnout improved. Finally, Zarate et al.'s (2019) meta-analysis also indicated that teaching mindfulness significantly reduced teacher burnout, thereby improving the wellbeing of the teacher population. In summary, the results of these different perspectives collectively indicate that mindfulness in teaching is an important influence on teacher burnout.

Teacher self-efficacy, derived from Bandura's (1986) social learning theory , refers to a teacher's subjective perception and judgment of their ability to influence educational outcomes, their own teaching levels, and their impact on children's learning and development (Hong and Pang, 2006). It includes a teacher's sense of efficacy in teaching strategies, classroom management, and student participation (Tschannen-Moran et al., 1998). Teacher self-efficacy also influences teachers' teaching attitudes, motivations, and behaviors (Wu and Zhan, 2017). A study by Yang and Hu (2021) on a group of teachers found that teachers with higher self-efficacy were more efficient in their teaching work. Similarly, teachers with lower self-efficacy will be less effective and spend more time and energy on their work (Jiang et al., 2023), which may correlate with a higher likelihood of burnout. For example, Wang and Li (2016) showed that preschool teachers with low self-efficacy experienced more negative emotions in their teaching work, and were dissatisfied with their current jobs and more likely to experience burnout. Hassan and Ibourk (2021) modeled the structural equation of teacher self-efficacy and burnout and found that self-efficacy was significantly negatively correlated with the emotional exhaustion of teacher burnout, low achievement, and depersonalization.

Previous studies have primarily utilized traditional structural equation modeling (SEM), treating teacher mindfulness, teacher burnout, and teacher self-efficacy as unitary constructs. These studies established mediation and moderation models to understand the relationships among these variables (Cheng et al., 2022; Hassan and Ibourk, 2021). However, these studies present several limitations. On the one hand, the majority of research employed cross-sectional designs, which can only demonstrate correlations between variables and cannot establish causality (He et al., 2024). For instance, Cheng et al. (2022) found that mindfulness in preschool teachers significantly and negatively predicted occupational burnout, with self-efficacy mediating the relationship between mindfulness and burnout. Ye et al. (2020) also developed a moderated mediation model to explore the effect of organizational justice on burnout. On the other hand, the theoretical models of teacher mindfulness and self-efficacy developed in Western research may not fully translate to the Chinese cultural context (Tschannen-Moran et al., 1998; Xanthopoulou et al., 2007). Chinese culture, with its emphasis on collectivism and interpersonal harmony (Dong, 2019), likely influences how these constructs are experienced and expressed. Western research often focuses on individual-level factors, such as teachers' personal coping strategies (Shapiro et al., 2005) and the impact of interventions like mindfulness training on teacher burnout (Taylor et al., 2021). In contrast, Chinese research tends to emphasize the sociocultural context, particularly the role of interpersonal relationships (e.g., teacher-student relationships, collegial support, family support) in teacher wellbeing and burnout (Li et al., 2020). Strained teacher-student relationships and a lack of collegial support, for example, are identified as significant contributors to burnout in Chinese teachers (Zhao et al., 2023). While mindfulness interventions, originating in the West, have been adopted in China, their effectiveness is moderated by cultural differences. Western mindfulness training typically prioritizes individual self-awareness, which contrasts with the Chinese emphasis on social support and interconnectedness (He et al., 2024). This difference may explain the limited effectiveness of some Westernized, self-focused mindfulness interventions in Chinese settings. To enhance their applicability, researchers are integrating mindfulness interventions with traditional Chinese cultural practices. For example, Wang et al. (2024) combined mindfulness with poetry, incorporating practices like poetry interpretation, listening, mindful writing, and recitation to cultivate mindfulness and wellbeing. This approach leverages the lyrical and philosophical depth of poetry within Chinese tradition, increasing its cultural resonance. Similarly, Gu et al. (2024) integrated mindfulness with group dance, encouraging participants to embody mindfulness through movement and promoting stress reduction through a combination of dynamic and static practices. Group dance, with its emphasis on interpersonal interaction and collective harmony, aligns well with Chinese collectivist values. These culturally adapted interventions offer several advantages, including increased cultural relevance, the incorporation of social support, and a focus on holistic wellbeing, making them more suitable for the psychological needs and cultural habits of Chinese individuals compared to more ego-centric Western approaches.

Network analysis offers a novel approach to understanding complex psychological and behavioral systems by representing and analyzing system characteristics through network structures (Borsboom et al., 2021; Epskamp et al., 2018a,b). By depicting interactions within a system as a network of “nodes” and “edges,” this method allows for the identification of node centrality and inter-node relationships. This approach facilitates the identification of the most influential variables within the network and prepares the ground for both exploratory/practice-oriented and theory-driven research (Chen, 2012).

First, network analysis reveals non-linear and interdependent relationships. Structural equation modeling (SEM) typically relies on assumptions of linearity and posits latent variables to explain the covariance among observed variables. These assumptions limit SEM's ability to capture complex non-linear relationships. In contrast, network analysis focuses directly on the conditional associations between observed variables without presupposing linearity or the existence of latent variables. This allows it to more effectively capture complex interdependencies, such as feedback loops and interactions, which are difficult to model within the SEM framework (Borsboom et al., 2021).

Second, a prominent feature of network analysis is its visualization capacity. It offers a clear visual representation of complex dynamics, illustrating nodes and their connections (Borsboom et al., 2021; Bringmann and Eronen, 2018). By employing partial correlations, network analysis controls for the influence of other nodes, providing a more accurate depiction of the true relationships between variables (Cramer et al., 2016). Importantly, network analysis can effectively explore the structure of high-dimensional data even in the absence of prior theoretical knowledge regarding variable associations (Borsboom et al., 2021). While SEM is widely used in theory-driven research, it has limitations when applied to complex psychological phenomena. Its reliance on predefined latent variables can introduce researcher bias and restrict the direct exploration of relationships among observed variables. Furthermore, SEM struggles to differentiate between genuine relationships and spurious correlations in cross-sectional data, a challenge addressed by network analysis through its ability to uncover “independent” relationships between variables. For instance, while studies using SEM have highlighted the importance of enhancing teacher self-efficacy in preventing burnout (e.g., Buzzai et al., 2024), their reliance on pre-specified theoretical models makes identifying optimal intervention targets challenging (Borsboom et al., 2021). Specifically, cross-sectional SEM studies struggle to unravel the complex dynamic relationships between variables, providing only correlational evidence and failing to capture the interactive processes within the system (Borsboom et al., 2021).

Finally, network analysis overcomes these limitations by incorporating all observed variables into a unified network framework, allowing for the examination of the emergence and development of psychological or behavioral systems from a holistic perspective (Cai et al., 2020). Its strength lies in revealing complex interactions and identifying key nodes with the greatest systemic impact, providing more precise targets for interventions. For example, Xie et al. (2022) utilized cross-lagged network analysis to explore the developmental trajectory of teacher burnout in primary school teachers and proposed effective intervention strategies. This demonstrates the advantage of network analysis in identifying optimal intervention targets for teacher burnout and informing the design of targeted interventions, a feat difficult to achieve with traditional cross-sectional SEM.

Therefore, this study employs network analysis to investigate the relationships between teaching mindfulness and teacher burnout, and between teacher self-efficacy and teacher burnout, offering advantages over traditional SEM. While SEM assumes linear relationships, network analysis captures the more complex and dynamic interplay between variables. Specifically, constructing a network model will facilitate the exploration of the most influential nodes and connections within the teaching mindfulness-teacher burnout and teacher self-efficacy-teacher burnout networks. By intervening on these core nodes and connections, we aim to mitigate the entire burnout network, rather than addressing only individual symptoms. Consequently, the network analysis approach is particularly suited to this study, providing a more comprehensive and nuanced understanding of teacher burnout and laying the foundation for more effective intervention strategies.

## 2 Materials and methods

### 2.1 Participants

In May 2024, a random sampling method was used to select kindergarten teachers from Suining City in Sichuan Province and Kunming City in Yunnan Province as the study subjects. A total of 610 paper questionnaires were randomly distributed. After excluding invalid data (e.g., incomplete, duplicate, or excessively patterned responses), 38 questionnaires were discarded, leaving 572 valid questionnaires entered into the EpiData 3.1 software, resulting in a response rate of 93.77%. Among the participants, 22 were male teachers, and 550 were female teachers. The age range was from 19 to 57 years (M = 24.46, SD = 5.35). In terms of teaching experience, 249 participants (43.6%) had less than 5 years of experience, 174 (30.4%) had 5 to 10 years, and 149 (26.0%) had more than 10 years. Regarding economic income, the monthly salaries of preschool teachers were distributed as follows: 179 teachers (31.3%) earned less than ¥4,000; 231 teachers (40.4%) earned between ¥4,001 and ¥6,000; 87 teachers (15.2%) earned between ¥6,001 and ¥8,000; and 75 teachers (13.1%) earned over ¥8,000. The teacher population in private kindergartens consisted of 160 individuals, representing 28% of the total sample. Conversely, public kindergartens employed 412 teachers, accounting for 72% of the total. Regarding location, 142 teachers worked in rural kindergartens, constituting 24% of the sample, while 430 teachers were employed in urban kindergartens, making up 75.2% of the sample. The educational background of the participants was as follows: 22 participants (3.8%) held an associate's degree, 386 participants (66.8%) held a bachelor's degree, and 164 participants (28.4%) held a graduate degree. Prior to completing the questionnaire, research staff provided a detailed explanation of the guidelines and precautions. This study was approved by the Ethics Committee of Sichuan Vocational and Technical College (Ethics Approval No: SCSXLXH2023052-1) and complies with the provisions of the Declaration of Helsinki.

### 2.2 Measurements

#### 2.2.1 Teaching mindfulness scale

This study used the Chinese version of the *Mindfulness in Teaching Scale* (MTS-C), developed by Frank et al. (2016) and translated and revised by Ma et al. (2021) for Chinese kindergarten teachers. The scale consists of 14 items across two dimensions: personal mindfulness (9 items) and interpersonal mindfulness (5 items). Personal mindfulness assesses teachers' mindfulness levels in the teaching environment, such as “Sometimes I get distracted while teaching, but I don't realize it at the time.” Interpersonal mindfulness evaluates teachers' mindfulness levels during teacher-student interactions, such as “I listen carefully to children's ideas, even if I disagree with them.” A Likert scale from 1 (never) to 5 (always) was used, with reverse scoring for the personal mindfulness dimension. After reversing the scores, a higher total score indicates a higher level of teaching mindfulness (both personal and interpersonal mindfulness) (Ma et al., 2021). The scale's Cronbach's alpha was 0.70, with personal mindfulness at 0.82 and interpersonal mindfulness at 0.74. Confirmatory factor analysis demonstrated adequate structural validity for the *Teaching Mindfulness Scale*, with CFI = 0.92, TLI = 0.90, and RMSEA = 0.06[90% CI, 0.05, 0.07]. These indices indicate a good model fit to the data, in accordance with the recommendations of Kline (2023).

#### 2.2.2 Teacher burnout scale

*The Teacher Burnout Scale* was developed by Zuo and Xi (2008). It features a total of 21 questions, consisting of three dimensions, and is scored on a 5-point Likert scale. There were 7 questions measuring low achievement (e.g., “It is easy for me to understand how young children are feeling,” which was scored on a reverse scale), 8 questions measuring emotional exhaustion (e.g., “My work drains me of my energy”), and 6 questions measuring depersonalization (e.g., “Since becoming a teacher I have become less and less affectionate toward young children”). Higher total scores for subjects indicate more severe burnout. In this study, the Cronbach's alpha for the Teacher Burnout Scale was 0.92, with emotional exhaustion at 0.90, depersonalization at 0.88, and low achievement at 0.86. Confirmatory factor analysis demonstrated good structural validity for the Teacher Self-Efficacy Scale, with CFI = 0.93, TLI = 0.92, and RMSEA = 0.08 [90% CI, 0.07, 0.09].

#### 2.2.3 Teacher self-efficacy scale

The Chinese version of the *Teacher Self-Efficacy Scale*, revised by Wu and Chim (2017), was used to measure the self-efficacy of preschool teachers. The scale consists of 12 items on a 5-point Likert scale, including two dimensions, four of which measured classroom management self-efficacy (e.g., “I am able to keep children in the classroom disciplined”), and 8 of which measured learning and teaching efficacy (e.g., “I am able to use a variety of teaching strategies in the classroom”), with higher scores on the scale indicating better levels of self-efficacy. The Cronbach's alpha for the *Teacher Self-Efficacy Scale* was 0.94, with classroom management self-efficacy at 0.86 and learning and teaching efficacy at 0.92. Confirmatory factor analysis demonstrated good structural validity for the *Teacher Burnout Scale*, with CFI = 0.90, TLI = 0.89, and RMSEA = 0.07 [90% CI, 0.06, 0.08].

### 2.3 Statistical analysis

First, SPSS 26.0 was used to calculate the means and standard deviations of nodes in the network. Next, the network model was constructed using the R package qgraph, and a Gaussian graphical model was used to fit the data (Epskamp et al., 2018b). As an undirected network, the edges of the Gaussian graphical model denote the partial correlation between two nodes, which is a net correlation between the two nodes after statistically controlling for the other nodes in the network (Epskamp et al., 2018b). Combining the graphical “least absolute shrinkage and selection operator” (LASSO) algorithm and the “extended Bayesian information criterion” (EBIC) for model selection yields a more stable and more interpretable network (Friedman et al., 2008). Spearman correlation was used as the input to the Gaussian graphical model, the hyperparameter of the EBIC was set to 0.5, and the network was laid out according to the Fruchterman-Reingold algorithm. Two network models were constructed, namely, the teaching mindfulness and teacher burnout network, and the self-efficacy and teacher burnout network. The nodes in the networks were predefined as two communities, namely, the teaching mindfulness community and the teacher burnout community in the teaching mindfulness and the teacher burnout network, and the self-efficacy community and the teacher burnout community in the self-efficacy and the teacher burnout network.

The evaluation of node bridge centrality was performed using the R package networktools (Jones et al., 2018). The bridge centrality metric selected for this study is BEI, which represents the sum of the edge weights of a node with nodes in other communities and is suitable for the assessment of networks that contain negative and positive associations, in which a higher bridge expected influence represents a higher degree of association with other communities (Jones et al., 2018). In this study, we constructed two network models.

Network robustness was tested using the R package bootnet (Epskamp et al., 2018a,b). The stability of the BEI was assessed using the case-dropping bootstrapping (1,000 bootstrapped samples) and using the correlation stability coefficient, which ideally should have a value greater than 0.5. We also conducted the edge accuracy test, the edge difference test, and the bridge centrality difference test.

## 3 Results

### 3.1 Descriptive statistics and correlation analysis

Tables 1 and 2 display the means, standard deviations, and BEIs of the variables in the teaching mindfulness-teacher burnout and teacher self-efficacy-teacher burnout networks, respectively. Table 3 presents the correlation analysis among variables, as well as the skewness and kurtosis of the data.

**Table 1:** The means, standard deviations, and BEIs of the variables in the teaching mindfulness-teacher burnout network (*n* = 572).

**Teaching mindfulness abbreviation**	**x ±s**	**BEI**
M1: Non-automated instruction	4.14 ± 0.75	−0.13
M2: Concentrate on the present moment	4.24 ± 0.82	−0.05
M3: Concentrate on teaching	4.22 ± 0.77	−0.06
M4: Focus on process	3.95 ± 0.84	−0.02
M5: Attention to experiences along the way	3.68 ± 1.01	−0.04
M6: Whole-body teaching	4.03 ± 0.86	−0.01
M7: Appropriate expression of pain	4.30 ± 0.92	−0.17
M8: Listening to young children's expressions	4.24 ± 0.75	−0.04
M9: Overcoming teaching difficulties	3.69 ± 0.95	−0.10
M10: Allowing young children to express themselves	3.61 ± 1.21	−0.01
M11: Listening to young children's opposing views	3.70 ± 1.22	−0.07
M12: Emotions affect young children	3.22 ± 1.22	−0.02
M13: Think twice before you act	2.74 ± 1.15	0.00
M14: Calmly dealing with young children	2.70 ± 1.23	−0.03
Teacher Burnout Factors	x ± s	BEI
J1: Emotional exhaustion	20.97 ± 6.71	−0.22
J2: Depersonalization	15.88 ± 4.01	−0.06
J3: Low achievement	16.43 ± 4.91	-−0.46

**Table 2:** The means, standard deviations, and BEIs of the variables in the teacher self-efficacy-teacher burnout network. (*n* = 572).

**Teacher self-efficacy abbreviation**	**x ±s**	**BEI**
S1: Stopping young children's disruptive behavior	3.56 ± 1.08	0.00
S2: Encouraging young children to love learning	3.73 ± 1.04	−0.08
S3: Getting young children to do well in school	3.74 ± 1.00	−0.01
S4: Engaging young children to value learning	3.45 ± 0.99	−0.05
S5: Ask good questions of young children in the classroom	3.47 ± 0.93	0.00
S6: Getting children to follow classroom discipline	3.53 ± 1.00	−0.03
S7: Keeping disruptive toddlers quiet	3.55 ± 1.00	0.00
S8: Establishing effective classroom management models	3.41 ± 1.00	−0.02
S9: Evaluating young children from multiple perspectives	3.60 ± 0.95	0.00
S10: Multiple explanations to help young children understand	3.73 ± 1.00	0.00
S11: Home-school cooperation to help young children	3.65 ± 0.98	−0.08
S12: Use multiple instructional strategies in the classroom	3.43 ± 0.96	−0.05
Teacher Burnout Factors	x ± s	BEI
J1: Emotional exhaustion	20.97 ± 6.71	0.00
J2: Depersonalization	15.88 ± 4.01	−0.02
J3: Low achievement	16.43 ± 4.91	−0.32

**Table 3:** Correlation analysis and skewness and kurtosis of the data. (*n* = 572).

	**M**	**SD**	**Skewness**	**Kurtosis**	**Correlation**		
					**1**	**2**	**3**
Teaching Mindfulness	52.45	6.27	0.09	0.1	1		
Teacher self-efficacy	42.85	9.28	−0.32	0.01	0.52^***^	1	
Teacher burnout	53.29	14.69	−0.23	2.03	−0.42^***^	−0.42^***^	1

^***^p < 0.001.

### 3.2 Common method bias

To test for common method bias, the Harman single-factor test was conducted. Eight factors had eigenvalues >1, and the first factor explained 27.69% of the variance, which is below the 40% threshold. This indicates that no significant common method bias is present (Tang and Wen, 2020).

### 3.3 Network analysis

#### 3.3.1 The network structures

**Figure 1:**
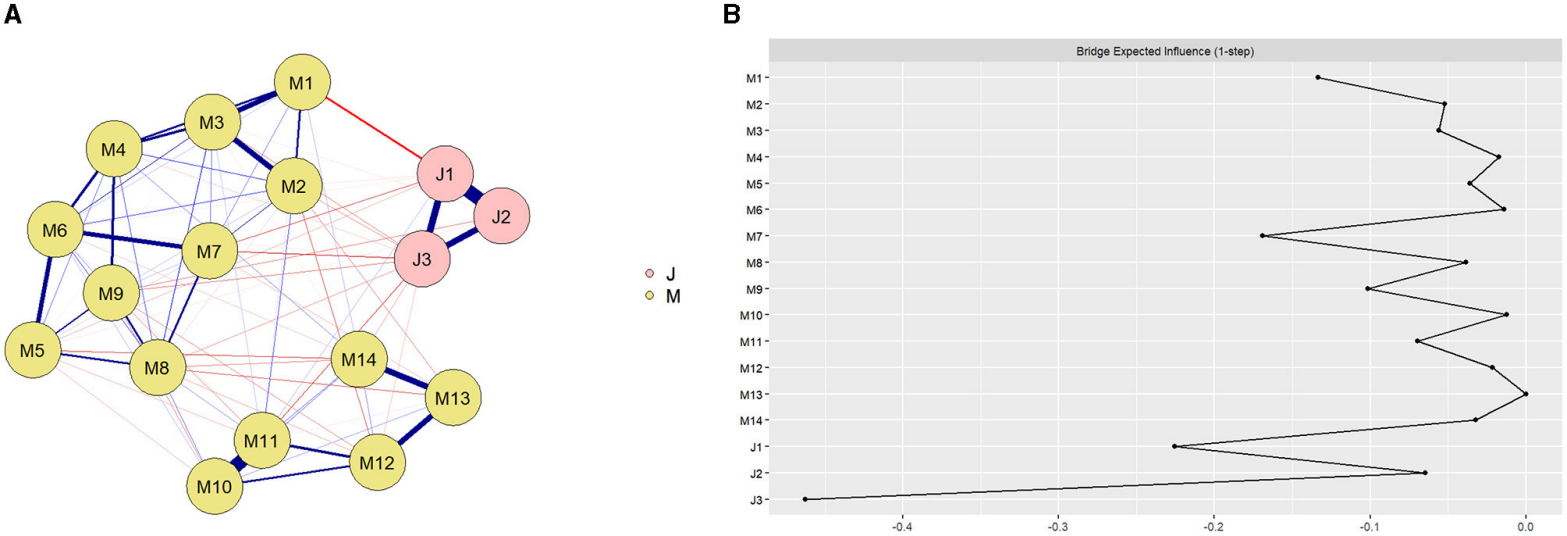
**(A)** Network structure of preschool teachers' teaching mindfulness and teacher burnout. Preschool teachers' teaching mindfulness (M1: Non-automated instruction, M2: Concentrate on the present moment, M3: Concentrate on teaching, M4: Focus on process, M5: Attention to experiences along the way, M6: Whole-body teaching, M7: Appropriate expression of pain, M8: Listening to young children's expressions, M9: Overcoming teaching difficulties, M10: Allowing young children to express themselves, M11: Listening to young children's opposing views, M12: Emotions affect young children, M13: Think twice before you act, M14: Calmly dealing with young children); and teacher burnout (J1: Emotional exhaustion, J2: Depersonalization, J3: Low achievement), with the blue line indicating a positive correlation and the red line indicating a negative correlation; the thicker the line and the more saturated the color, the stronger the correlation. See Appendix 11 for details. (**B)** The values of the bridge expected influence for each node in the network. Teaching mindfulness (M1: Non-automated instruction, M2: Concentrate on the present moment, M3: Concentrate on teaching, M4: Focus on process, M5: Attention to experiences along the way, M6: Whole-body teaching, M7: Appropriate expression of pain, M8: Listening to young children's expressions, M9: Overcoming teaching difficulties, M10: Allowing young children to express themselves, M11: Listening to young children's opposing views, M12: Emotions affect young children, M13: Think twice before you act, M14: Calmly dealing with young children), and teacher burnout (J1: Emotional exhaustion, J2: Depersonalization, J3: Low achievement). See Appendix 12 for details.

The network structure of teaching mindfulness and teacher burnout among preschool teachers (Figure 1A) showed that there were 20 association paths across teaching mindfulness and teacher burnout, of which 19 were negatively correlated and 1 was positively correlated, with edge weights ranging from −0.13 to 0.03. J1, “Emotional exhaustion,” was negatively correlated with five nodes of teaching mindfulness, with the strongest negative correlation being M1, “Non-automated instruction,” with an edge weight of −0.13. However, J1, “Emotional exhaustion,” was positively correlated with M11, “Listening to young children's opposing views,” with an edge weight of 0.03. J2, “Depersonalization,” was negatively correlated with 3 nodes of teaching mindfulness, and was most negatively correlated with M9, “Overcoming teaching difficulties,” with an edge weight of−0.05. J3, “Low achievement,” was negatively correlated with 11 nodes of teaching mindfulness and was most negatively correlated with M7, “Appropriate expression of pain.” The associations within the teaching mindfulness community were mostly positive, with the strongest associations being between M10, “Allowing young children to express themselves,” and M11, “Listening to young children's opposing views,” with edge weights of 0.55. The associations within the teacher burnout community were mostly positive, with the strongest associations being between J1, “Emotional exhaustion,” and J2, “Depersonalization,” with an edge weight of 0.56. The specific values of all edge weights in the network are shown in Appendix 1.

**Figure 2:**
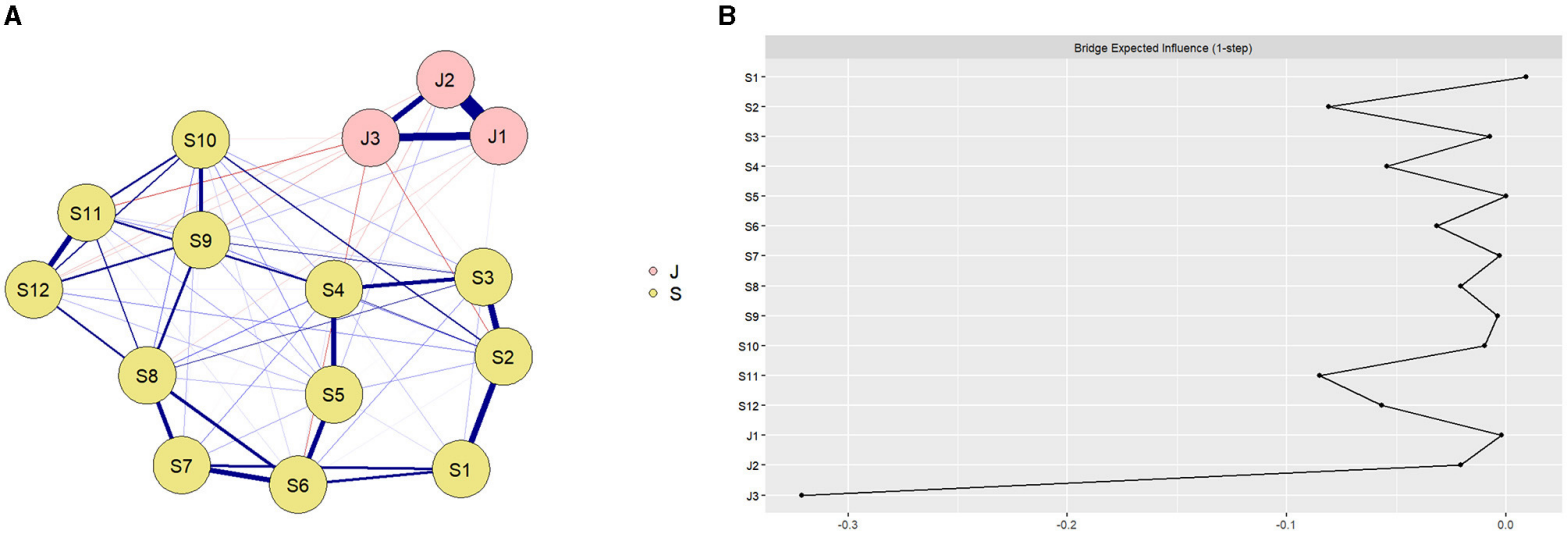
**(A)** Network structure of preschool teachers' self-efficacy and teacher burnout. Preschool teachers' self-efficacy (S1: Stopping young children's disruptive behavior, S2: Encouraging young children to love learning, S3: Getting young children to do well in school, S4: Engaging young children to value learning, S5: Ask good questions of young children in the classroom, S6: Getting children to follow classroom discipline, S7: Keeping disruptive toddlers quiet, S8: Establishing effective classroom management models, S9: Evaluating young children from multiple perspectives, S10: Multiple explanations to help young children understand, S11: Home-school cooperation to help young children, S12: Use multiple instructional strategies in the classroom); and teacher burnout (J1: Emotional exhaustion, J2: Depersonalization, J3: Low achievement), with the blue line indicating a positive correlation and the red line indicating a negative correlation; the thicker the line and the more saturated the color, the stronger the correlation. See Appendix 13 for details. **(B)** The values of the bridge expected influence for each node in the network. Teacher self-efficacy (S1: Stopping young children's disruptive behavior, S2: Encouraging young children to love learning, S3: Getting young children to do well in school, S4: Engaging young children to value learning, S5: Ask good questions of young children in the classroom, S6: Getting children to follow classroom discipline, S7: Keeping disruptive toddlers quiet, S8: Establishing effective classroom management models, S9: Evaluating young children from multiple perspectives, S10: Multiple explanations to help young children understand, S11: Home-school cooperation to help young children, S12: Use multiple instructional strategies in the classroom); and teacher burnout (J1: Emotional exhaustion, J2: Depersonalization, J3: Low achievement). See Appendix 14 for details.

The network structure of teacher self-efficacy and teacher burnout among preschool teachers (Figure 2A) showed that there were 16 association paths across teacher self-efficacy and teacher burnout, of which 13 were negatively correlated and 3 were positively correlated, with edge weights ranging from −0.09 to 0.03. J1, “Emotional exhaustion,” was negatively correlated with 3 nodes of teacher self-efficacy, with the strongest negative correlation being with S4, “Engaging young children to value learning,” with an edge weight of −0.02. J2, “Depersonalization,” was negatively correlated with 2 nodes of teacher self-efficacy, and was most strongly correlated with S4, “Engaging young children to value learning,” with an edge weight of −0.03. J3, “Low achievement,” was negatively correlated with 8 nodes of teaching mindfulness, and was most strongly correlated with S2, “Encouraging young children to love learning,” with an edge weight of −0.08. The associations within the teacher self-efficacy community were all positive, with the strongest association between S2, “Encouraging young children to love learning,” and S3, “Getting young children to do well in school,” with an edge weight of 0.32. The associations within the teacher burnout community were all positive, with J1, “Emotional exhaustion,” and S3, “Getting young children to do well in school,” being the strongest associations, with an edge weight of 0.57. The specific values of all edge weights in the network are shown in Appendix 2.

#### 3.3.2 The bridge expected influences (BEI)

The results of the BEI in the teaching mindfulness-teacher burnout network for preschool teachers (Figure 1B) showed that the bridge expected influence of M1, “Non-automated instruction,” and M7, “Appropriate expression of pain,” were highest in the teaching mindfulness community, with values of −0.13 and −0.17, respectively. The fact that M1 and M7 are negatively associated with a wider range of teacher burnout symptoms suggests a potential protective relationship against teacher burnout. The correlation stability coefficient of BEI in the network was 0.65, which, being larger than 0.5 indicates sufficient stability (see Appendix 3 for details). The 95% bootstrapping confidence intervals around edge weights were small to moderate (see Appendix 4 for details), which revealed that the accuracy of the edges was acceptable. Edge weight difference tests and BEI difference tests are presented in Appendices 5 and 6.

The results of the BEI of teacher the self-efficacy-teacher burnout network for preschool teachers (Figure 2B) showed that the BEIs of S2, “Encouraging young children to love learning,” and S11, “Home-school cooperation to help young children,” had the highest values, −0.08 and −0.09, respectively, in the teacher self-efficacy community. The fact that S2 and S11 are negatively associated with a wider range of teacher burnout symptoms suggests a potential protective relationship against teacher burnout. The correlation stability coefficient of BEI in the network was 0.65, which, being larger than 0.5, indicates sufficient stability (see Appendix 7 for details). The 95% bootstrapping confidence intervals around edge weights were small to moderate (see Appendix 8 for details), which revealed that the accuracy of the edges was acceptable. Edge weight difference tests and BEI difference tests are presented in Appendices 9 and 10.

## 4 Discussion

The results of the network analyses indicated different patterns of associations between teacher burnout and teaching mindfulness or teacher self-efficacy.

First, there was a strong negative association between emotional exhaustion and teaching mindfulness M1, “Non-automated instruction,” which suggests a higher protective effect against emotionally draining burnout. For example, Saloviita and Pakarinen (2021) also found that teachers who were fully engaged in teaching were better supported by their students and reduced their own burnout. There was also a strong negative association between emotionally draining burnout and S4, “Engaging young children to value learning,” which suggests a strong protective effect against emotionally draining burnout. For example, Schaack et al. (2020) found that preschool teachers who taught challenging young children were able to achieve job satisfaction and thus reduce their own emotional burnout.

This study found a positive correlation “Emotional Exhaustion” (J1) and “Listening to Young Children's Opposing Views” (M11), a counterintuitive finding that warrants further investigation. Firstly, the high intensity of teacher-child interactions, especially when children express dissent, may be a significant contributor to teacher emotional exhaustion. Children's dissent can potentially increase teacher stress and emotional burden. Research by Bianchi and Schonfeld (2023) has demonstrated that frequently addressing student behavioral problems significantly increases teacher stress and emotional exhaustion. Furthermore, cultural context may also play a role. In Chinese culture, teachers are generally expected to maintain classroom harmony and order (Wang, 2006). Consequently, children expressing dissent might be perceived as a challenge to classroom order, increasing teacher stress and potentially being interpreted as a reflection of the teacher's inadequacy. Li et al. (2023) found that Chinese teachers are more inclined to adopt authoritarian strategies when dealing with student conflict, which may further exacerbate teacher stress when faced with young children's dissent. Secondly, the lack of classroom structure and support systems may also contribute to teacher emotional exhaustion. A well-organized classroom with clear rules and procedures can create a more manageable environment for handling dissenting opinions (Marzano et al., 2021). Conversely, a lack of structure can lead to chaos and stress, making teachers more susceptible to emotional exhaustion when confronted with dissent (Ansari et al., 2020). Moreover, support from colleagues, school leadership, and parents is crucial, acting as a buffer against work-related challenges, including managing young children's dissent, a lack of support can exacerbate teacher stress and emotional burden (Greenglass et al., 2020). In conclusion, the positive correlation between young children's dissent and teacher emotional exhaustion is a complex issue influenced by multiple factors. Future research should further explore this relationship, focusing on classroom management strategies, cultural context, and the role of support systems to develop more effective interventions to enhance early childhood teachers' wellbeing.

Second, there is a strong negative correlation between depersonalization and teaching mindfulness M9, “Overcoming teaching difficulties,” which suggests that this factor has a strong protective effect on depersonalized burnout. For example, a follow-up study by Skaalvik and Skaalvik (2020) found that teachers' efforts to cope with teaching stress and overcome difficulties were effective in reducing their burnout and enabling them to use their initiative in teaching tasks. Depersonalization burnout was also strongly negatively correlated with teacher self-efficacy S4, “Engaging young children to value learning,” which suggests a strong protective effect against depersonalization burnout. For example, a meta-analysis by Park and Shin (2020) also indicated that teaching-related factors such as the age and gender of the early childhood educator (ECE) and the children's learning ability influence the level of depersonalization burnout among ECE teachers.

Next, there is a strong negative association between low achievement and teaching mindfulness M7, “Appropriate expression of pain,” which means that it has a certain protective effect on low-achievement burnout. For example, Bakker and de Vries (2021) found that when individuals face greater pressure at work, they can adopt appropriate strategies, such as taking the initiative to talk to others, which may be associated with better fatigue regulation and lower burnout. Low-achievement burnout was strongly and negatively associated with teachers' self-efficacy S2, “Encouraging young children to love learning,” and S3, “Getting young children to do well in school,” which means that these aspects have a protective effect on low-achievement burnout. For example, a meta-analysis by Park and Shin (2020) also indicated that the student factor of preschool performance affects preschool teachers' sense of achievement, specifically the sense of helplessness that preschool teachers feel when their pupils exhibit problematic behaviors and have low academic achievement.

The bridging expected influence (BEI) of nodes in the teaching mindfulness or teacher self-efficacy network may indicate the protective role of different aspects of teaching mindfulness or self-efficacy in mitigating teacher burnout (Gordesli, 2022). Targeting M7 (“appropriate expression of pain”) refers to kindergarten teachers recognizing and accepting their negative emotions (e.g., stress, anxiety, frustration) at work and expressing them in a healthy manner. Expressing distress can facilitate emotional regulation and the use of coping strategies, which was associated with lower levels of burnout (Shanafelt et al., 2015). Specifically, appropriately expressing distress can reduce emotional suppression, a known risk factor for occupational burnout (Scheibe and Moghimi, 2021). By expressing distress, teachers can release negative emotions, preventing emotional accumulation and outbursts (Maslach and Leiter, 2016), and gain social support and understanding (Khan et al., 2021). Furthermore, expressing distress can also help teachers better understand their own emotions and needs, enabling them to adopt more targeted coping measures (Salmela-Aro et al., 2019), such as seeking help, adjusting work methods, or seeking psychological counseling (Hascher and Waber, 2021). These coping strategies can enhance teachers' coping abilities and resilience, thereby reducing the risk of burnout (Agyapong et al., 2023). Additionally, a supportive work environment can buffer the negative effects of stress, and inviting psychological counselors to provide emotional support and stress management training can be beneficial (Maslach and Leiter, 2016). Finally, peer support is also an effective coping strategy, allowing teachers to share experiences, provide mutual encouragement, and offer emotional support (Herman et al., 2018).

Targeting S11 (“home-school cooperation to help young children”) underscores the critical role of strong, collaborative partnerships between teachers and parents in supporting children's holistic development. Positive family-school partnerships cultivate a more nurturing and effective learning environment (Epstein et al., 2018). Therefore, to promote holistic child development and enhance teacher professionalism, teacher training programs should effectively integrate mindfulness training and home-school collaboration strategies. Regarding mindfulness training, the programs should encompass practical skills such as focused breathing, mindful listening, and mindful eating, integrated into teaching activities (Lu et al., 2019). Furthermore, stress-reduction techniques incorporating traditional Chinese culture, such as Tai Chi, calligraphy, and tea ceremonies, should be included (Jin and Liu, 2017; Sun and Wang, 2020), adapting successful models like Mindful Schools to the local context (DeMauro et al., 2019). For home-school collaboration, training should cover the establishment of communication platforms (e.g., standardized use and procedures for WeChat groups and school-family communication apps), planning parent-child activities (Kang et al., 2019) such as organizing family sports days, reading activities, and craft workshops, and home visit techniques (Zhang, 2022), including mindful listening and respecting parents' cultural backgrounds. Additionally, the application of Epstein's parent involvement model should be incorporated (Zhou, 2015), using case studies and discussions to explore ways to integrate different types of parental involvement. Through these specific strategies, early childhood teachers can more effectively facilitate the physical and mental development of young children. In summary, targeting M7 and S11 requires the joint efforts of kindergartens, education authorities, and teacher training institutions to provide comprehensive support for kindergarten teachers.

This innovative study explores the mechanisms through which teaching mindfulness and teacher self-efficacy influence kindergarten teachers' burnout. However, future research should further investigate the interplay between teacher burnout and other mental health issues, such as anxiety and teacher burnout (Agyapong et al., 2022). Furthermore, because this study relied on self-report measures, potentially introducing social desirability bias, future research should incorporate multiple assessment methods (e.g., observational data of mindfulness interventions) to mitigate such biases (Taylor et al., 2021). Moreover, the cross-sectional design limits causal inferences; therefore, longitudinal designs (e.g., cross-lagged panel analysis) are recommended for future studies to examine changes over time (Xie et al., 2022). And, this study is limited to two provinces in Southwest China, and the generalizability of its findings may be affected by regional variations. For example, eastern and northern provinces differ from the southwest in terms of economic development, educational structure, and cultural traditions, potentially leading to variations in teachers' salaries and working conditions, competitive pressures, teaching requirements, home-school collaboration mechanisms, and coping strategies (Fu and Yao, 2018). Future research should expand the sample to include preschool teachers from diverse regions and backgrounds, incorporating variables such as economic development and educational structure as controls. Finally, given the gender imbalance in the sample (predominantly female), its generalizability to male kindergarten teachers is limited; thus, future research should prioritize a more balanced sample.

## 5 Conclusion

This study used network analysis to explore the relationship between preschool teachers' teaching mindfulness and teacher burnout, and the findings suggest that teaching mindfulness and self-efficacy, such as M7, “Appropriate expression of pain,” and S11, “Home-school cooperation to help young children,” may be potential targets for teacher burnout interventions. Identifying these key and bridge symptoms may provide guidance for burnout prevention for preschool teachers. In addition, by examining the link between teaching mindfulness or teacher self-efficacy and burnout, it may be possible to prevent the worsening of coexisting burnout problems in preschool teachers, and thus achieve more effective and comprehensive intervention for burnout prevention.

## Data Availability

The datasets presented in this study can be found in online repositories. The names of the repository/repositories and accession number(s) can be found at: https://osf.io/exkf9.
