# Supplementary material for: Exploring the interplay of mindfulness, self-efficacy, and burnout among Chinese preschool teachers: a network approach
Source: Front Psychol. 2025 Mar 12;16:1483099. doi: 10.3389/fpsyg.2025.1483099 (PMC11937131; doi:10.3389/fpsyg.2025.1483099)
Supplement: Supplementary file 1 [file Data_Sheet_1.pdf]

Appendix1

	M1	M2	M3	M4	M5	M6	M7	M8	M9	M10	M11	M12	M13	M14	J1	J2	J3
M1	0.0000	0.1344	0.2493	0.1508	0.0000	0.0243	0.0504	0.0000	0.0000	0.0000	0.0089	0.0000	0.0000	0.0261	-0.1334	0.0000	0.0000
M2	0.1344	0.0000	0.2678	0.0836	0.0000	0.0772	0.1064	0.0116	0.0229	0.0000	0.0720	-0.0660	-0.0466	0.0000	-0.0101	0.0000	-0.0420
M3	0.2493	0.2678	0.0000	0.1684	0.0031	0.1008	0.0588	0.0984	0.0000	0.0000	0.0217	0.0000	0.0000	-0.0049	0.0000	-0.0076	-0.0482
M4	0.1508	0.0836	0.1684	0.0000	0.0556	0.1743	0.0000	0.0688	0.1659	0.0000	0.0000	0.0000	0.0000	0.0479	0.0000	0.0000	-0.0173
M5	0.0000	0.0000	0.0031	0.0556	0.0000	0.2291	0.0165	0.1517	0.1236	-0.0258	-0.0267	0.0000	0.0000	-0.0779	-0.0281	-0.0079	0.0000
M6	0.0243	0.0772	0.1008	0.1743	0.2291	0.0000	0.2283	0.0751	0.0445	0.0267	0.0117	0.0000	0.0000	-0.0209	-0.0069	0.0000	-0.0070
M7	0.0504	0.1064	0.0588	0.0000	0.0165	0.2283	0.0000	0.1385	0.0233	0.0000	0.0000	0.0000	-0.0251	0.0000	-0.0727	0.0000	-0.0963
M8	0.0000	0.0116	0.0984	0.0688	0.1517	0.0751	0.1385	0.0000	0.1310	0.0538	0.0404	-0.0311	-0.0673	-0.0476	0.0000	0.0000	-0.0385
M9	0.0000	0.0229	0.0000	0.1659	0.1236	0.0445	0.0233	0.1310	0.0000	-0.0453	-0.0446	-0.0439	0.0000	0.0000	0.0000	-0.0488	-0.0526
M10	0.0000	0.0000	0.0000	0.0000	-0.0258	0.0267	0.0000	0.0538	-0.0453	0.0000	0.5506	0.1414	0.0366	0.0590	0.0000	0.0000	-0.0123
M11	0.0089	0.0720	0.0217	0.0000	-0.0267	0.0117	0.0000	0.0404	-0.0446	0.5506	0.0000	0.1699	0.0068	0.0601	0.0256	0.0000	-0.0950
M12	0.0000	-0.0660	0.0000	0.0000	0.0000	0.0000	0.0000	-0.0311	-0.0439	0.1414	0.1699	0.0000	0.2778	0.0440	0.0000	0.0000	-0.0216
M13	0.0000	-0.0466	0.0000	0.0000	0.0000	0.0000	-0.0251	-0.0673	0.0000	0.0366	0.0068	0.2778	0.0000	0.2997	0.0000	0.0000	0.0000
M14	0.0261	0.0000	-0.0049	0.0479	-0.0779	-0.0209	0.0000	-0.0476	0.0000	0.0590	0.0601	0.0440	0.2997	0.0000	0.0000	0.0000	-0.0321
J1	-0.1334	-0.0101	0.0000	0.0000	-0.0281	-0.0069	-0.0727	0.0000	0.0000	0.0000	0.0256	0.0000	0.0000	0.0000	0.0000	0.5555	0.3319
J2	0.0000	0.0000	-0.0076	0.0000	-0.0079	0.0000	0.0000	0.0000	-0.0488	0.0000	0.0000	0.0000	0.0000	0.0000	0.5555	0.0000	0.2949
J3	0.0000	-0.0420	-0.0482	-0.0173	0.0000	-0.0070	-0.0963	-0.0385	-0.0526	-0.0123	-0.0950	-0.0216	0.0000	-0.0321	0.3319	0.2949	0.0000

Appendix2

	S1	S2	S3	S4	S5	S6	S7	S8	S9	S10	S11	S12	J1	J2	J3
S1	0.0000	0.3008	0.0303	0.0309	0.0181	0.1692	0.1665	0.0000	0.0000	0.0025	0.0000	0.0000	0.0089	0.0000	0.0000
S2	0.3008	0.0000	0.3156	0.1043	0.0453	0.0068	0.0000	0.0000	0.0717	0.1172	0.0000	0.0585	0.0000	0.0000	-0.0808
S3	0.0303	0.3156	0.0000	0.2182	0.0031	0.0606	0.0000	0.1026	0.1008	0.0472	0.0250	0.0000	0.0000	0.0000	-0.0074
S4	0.0309	0.1043	0.2182	0.0000	0.2611	0.0140	0.0708	0.0780	0.0000	0.0600	0.1499	0.0081	-0.0237	-0.0306	0.0000
S5	0.0181	0.0453	0.0031	0.2611	0.0000	0.2472	0.0420	0.0348	0.0435	0.0618	0.0525	0.0358	0.0000	0.0000	0.0000
S6	0.1692	0.0068	0.0606	0.0140	0.2472	0.0000	0.2629	0.1935	0.0000	0.0216	0.0150	0.0065	0.0000	0.0377	-0.0693
S7	0.1665	0.0000	0.0000	0.0708	0.0420	0.2629	0.0000	0.2279	0.0048	0.0356	0.1309	0.0000	0.0000	0.0000	-0.0030
S8	0.0000	0.0000	0.1026	0.0780	0.0348	0.1935	0.2279	0.0000	0.1686	0.0694	0.0676	0.1399	-0.0207	0.0000	0.0000
S9	0.0000	0.0717	0.1008	0.0000	0.0435	0.0000	0.0048	0.1686	0.0000	0.2180	0.0573	0.1527	0.0378	0.0000	-0.0418
S10	0.0025	0.1172	0.0472	0.0600	0.0618	0.0216	0.0356	0.0694	0.2180	0.0000	0.1376	0.1199	0.0000	0.0000	-0.0097
S11	0.0000	0.0000	0.0250	0.1499	0.0525	0.0150	0.1309	0.0676	0.0573	0.1376	0.0000	0.2510	0.0000	0.0000	-0.0850
S12	0.0000	0.0585	0.0000	0.0081	0.0358	0.0065	0.0000	0.1399	0.1527	0.1199	0.2510	0.0000	-0.0044	-0.0280	-0.0242
J1	0.0089	0.0000	0.0000	-0.0237	0.0000	0.0000	0.0000	-0.0207	0.0378	0.0000	0.0000	-0.0044	0.0000	0.5779	0.3562
J2	0.0000	0.0000	0.0000	-0.0306	0.0000	0.0377	0.0000	0.0000	0.0000	0.0000	0.0000	-0.0280	0.5779	0.0000	0.2903
J3	0.0000	-0.0808	-0.0074	0.0000	0.0000	-0.0693	-0.0030	0.0000	-0.0418	-0.0097	-0.0850	-0.0242	0.3562	0.2903	0.0000

Appendix3

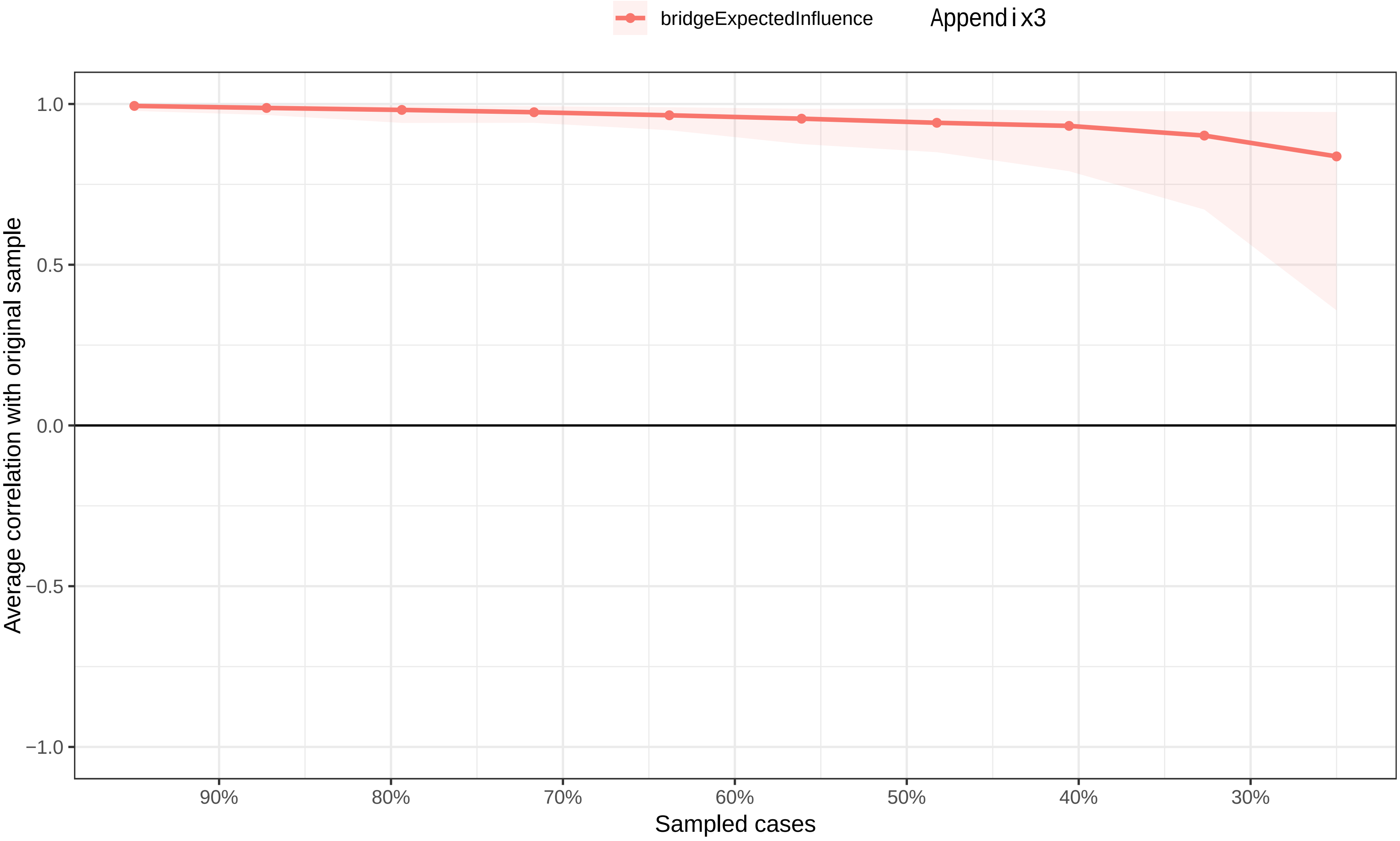

bridgeExpectedInfluence
1.0
0.5
0.0
−0.5
−1.0
Average correlation with original sample
90%
80%
70%
60%
50%
40%
30%
Sampled cases

Appendix4

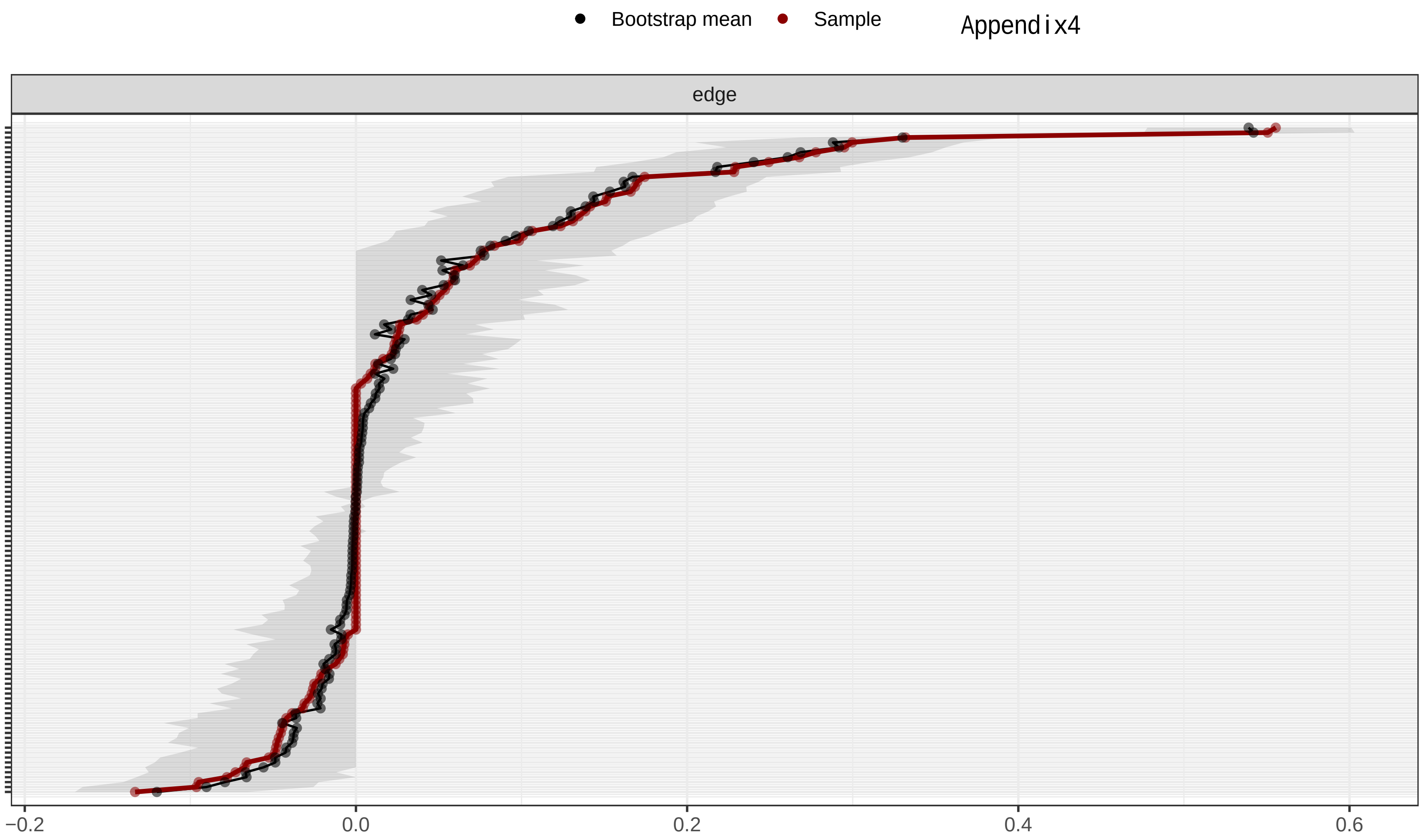

Appendix5

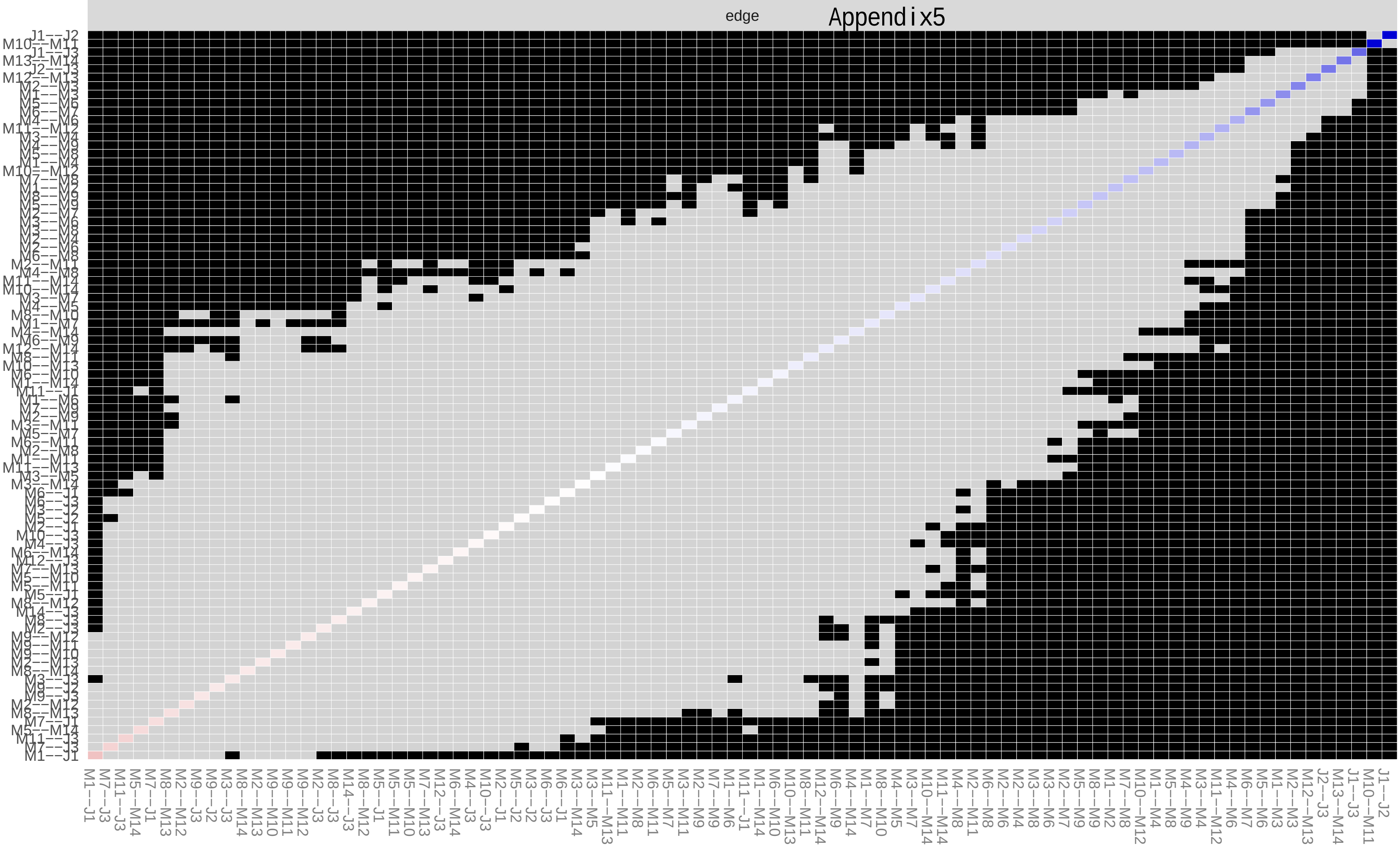

Appendix6

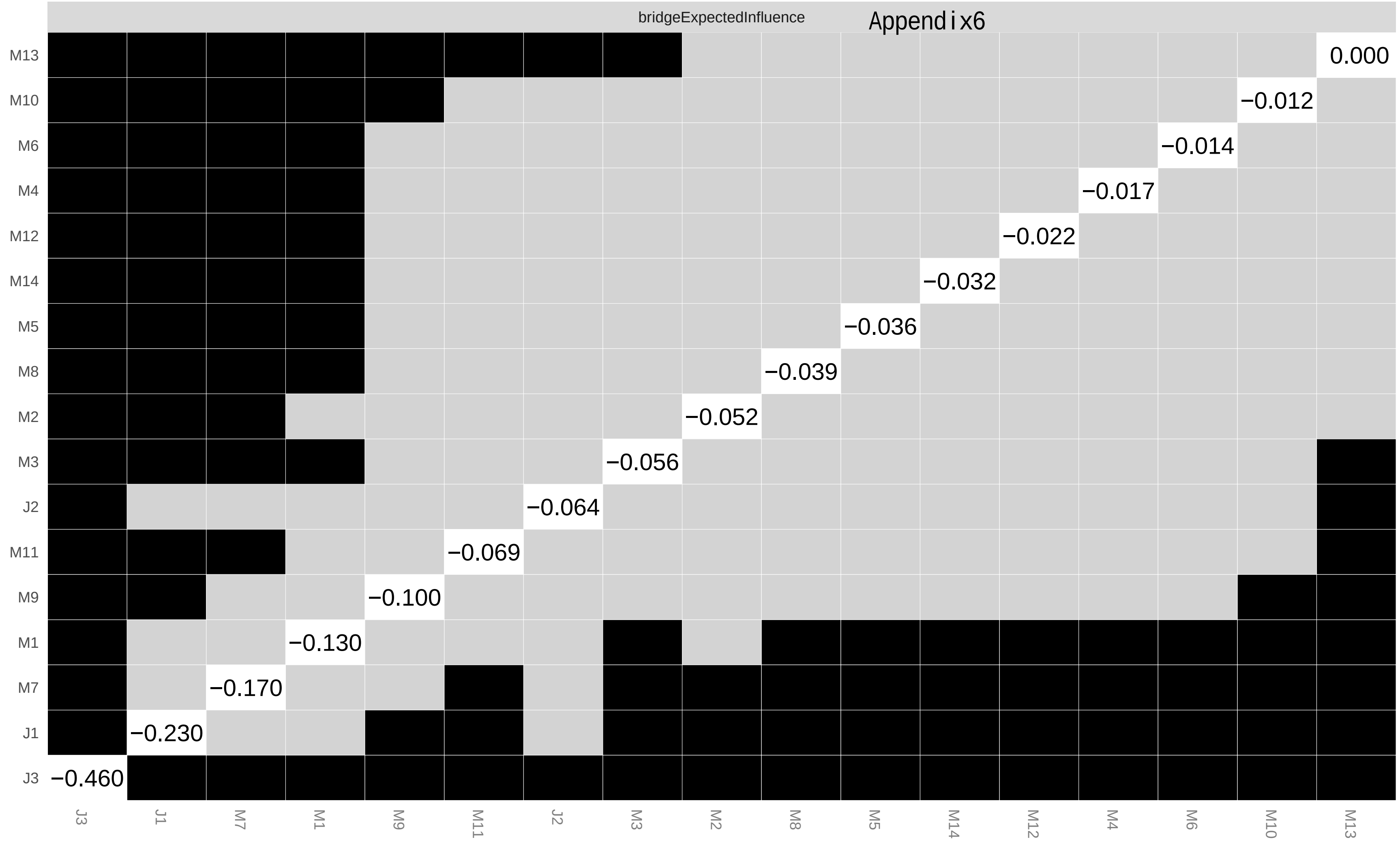
bridgeExpectedInfluence
M13
M10
M6
M4
M12
M14
M5
M8
M2
M3
J2
M11
M9
M1
M7
J1
J3
0.000
−0.012
−0.014
−0.017
−0.022
−0.032
−0.036
−0.039
−0.052
−0.056
−0.064
−0.069
−0.100
−0.130
−0.170
−0.230
−0.460
J3
J1
M7
M1
M9
M11
J2
M3
M2
M8
M5
M14
M12
M4
M6
M10
M13

Appendix7

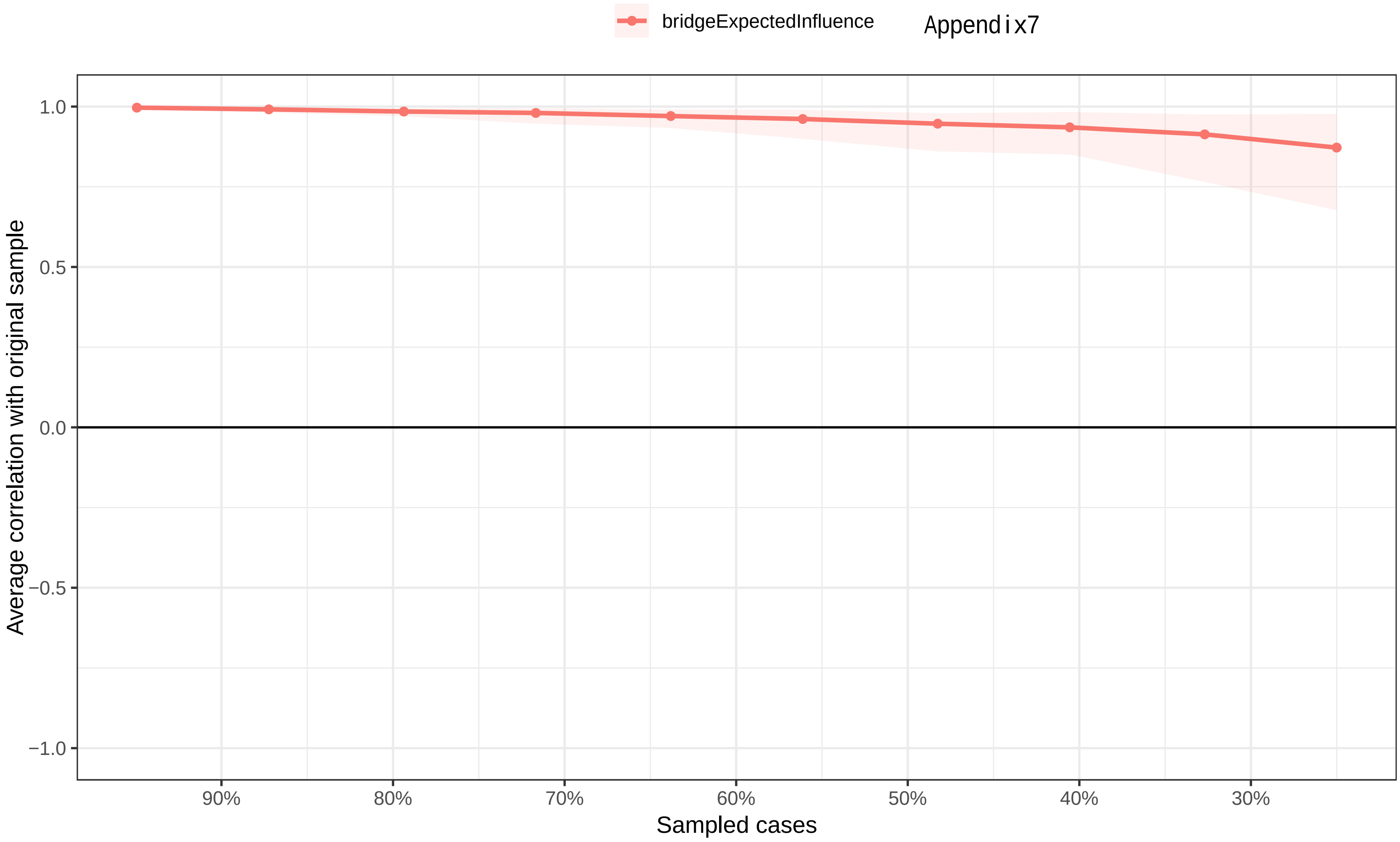
bridgeExpectedInfluence
Average correlation with original sample
1.0
0.5
0.0
−0.5
−1.0
90%
80%
70%
60%
50%
40%
30%
Sampled cases

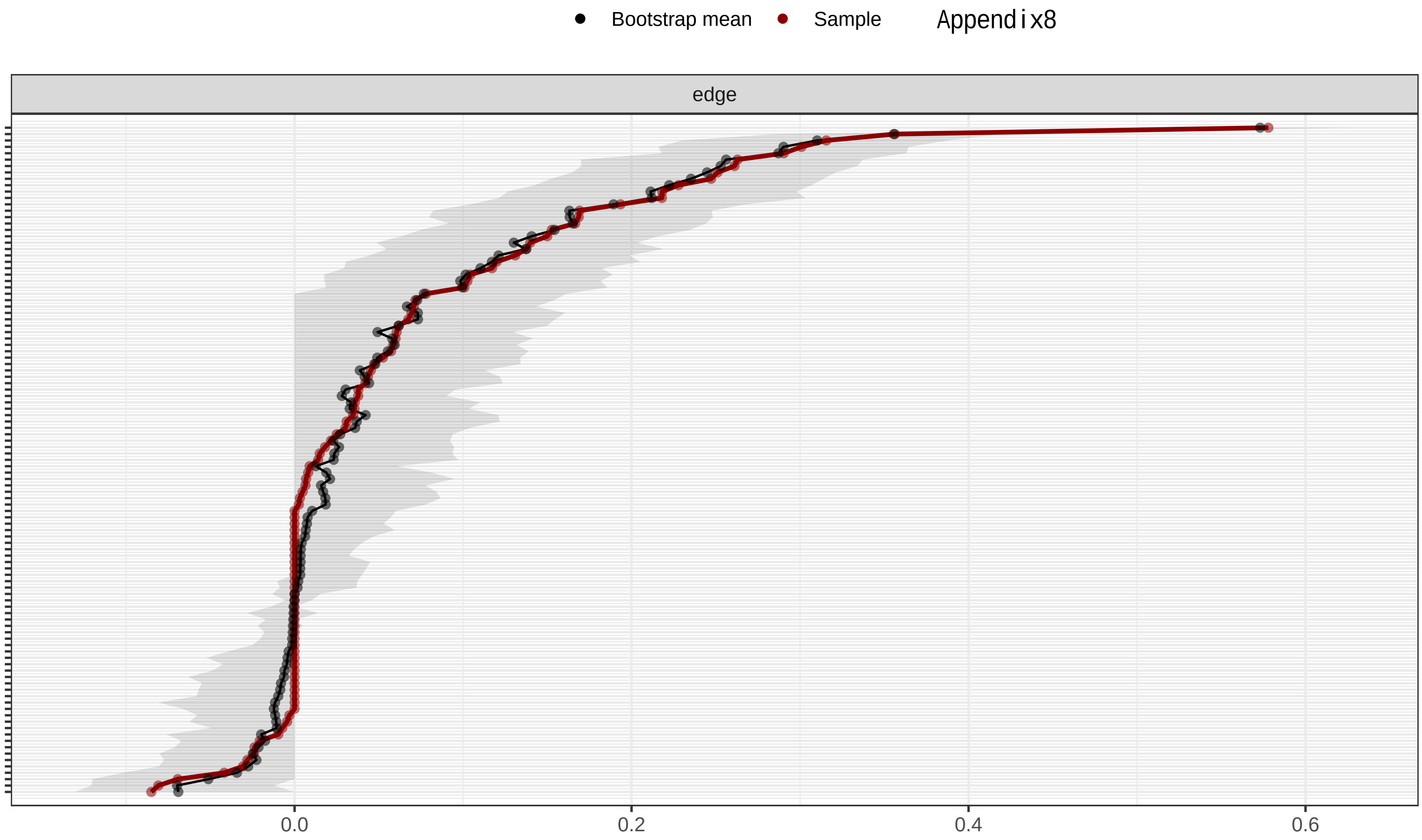

Bootstrap mean
Sample
edge
0.0
0.2
0.4
0.6

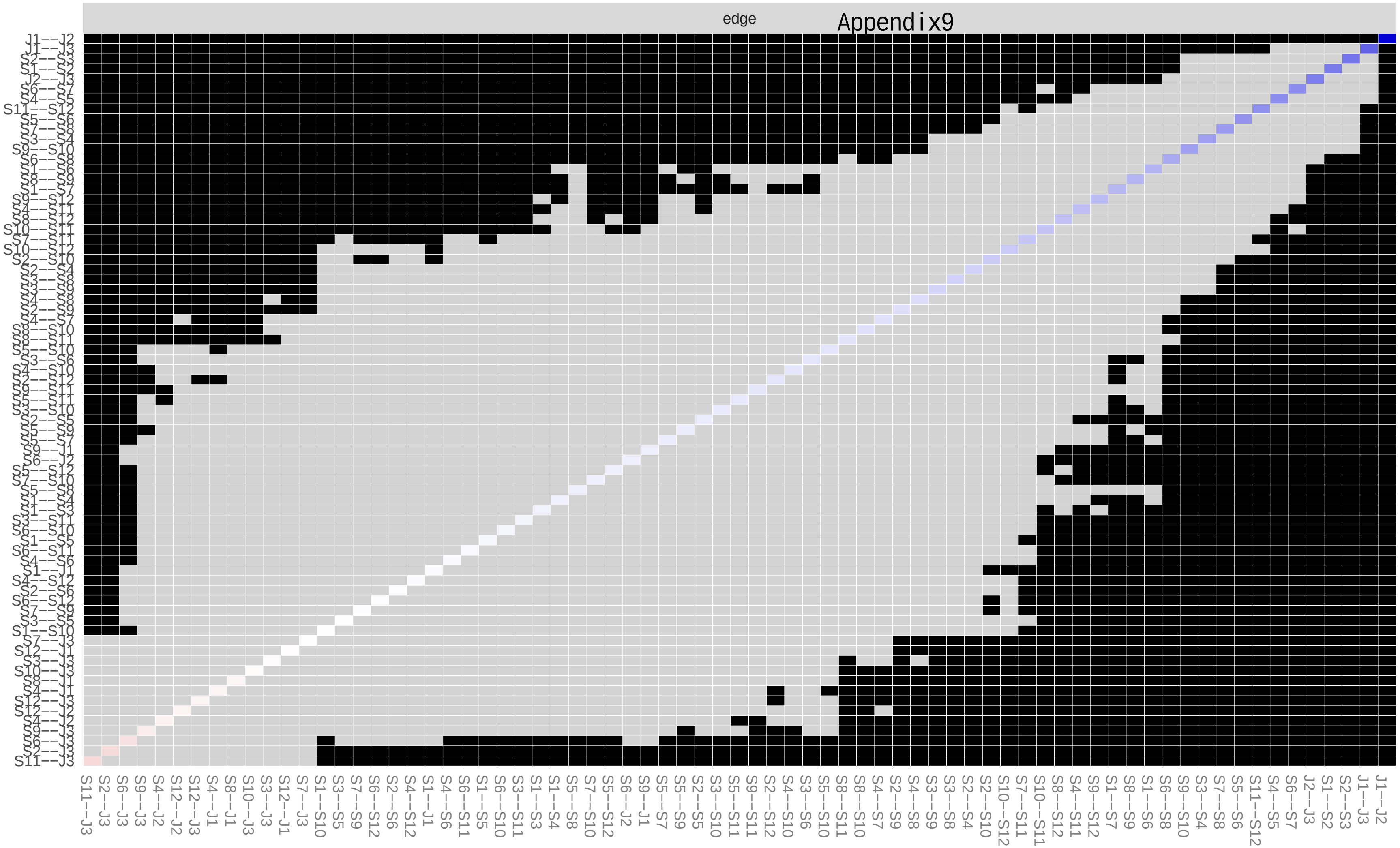

Appendix9
edge

Appendix10

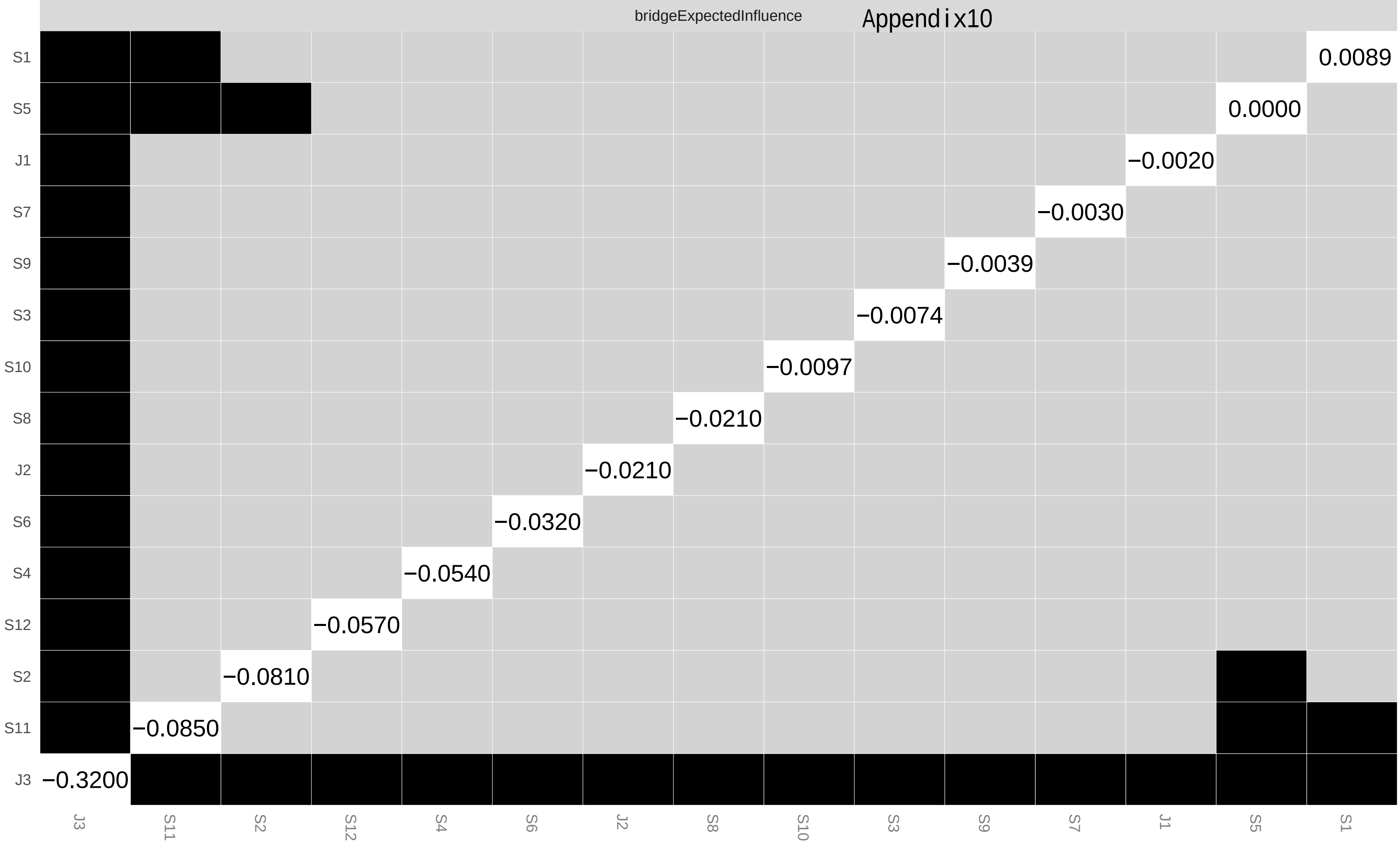
bridgeExpectedInfluence
S1
S5
J1
S7
S9
S3
S10
S8
J2
S6
S4
S12
S2
S11
J3
0.0089
0.0000
−0.0020
−0.0030
−0.0039
−0.0074
−0.0097
−0.0210
−0.0210
−0.0320
−0.0540
−0.0570
−0.0810
−0.0850
−0.3200
J3
S11
S2
S12
S4
S6
J2
S8
S10
S3
S9
S7
J1
S5
S1

Appendix11

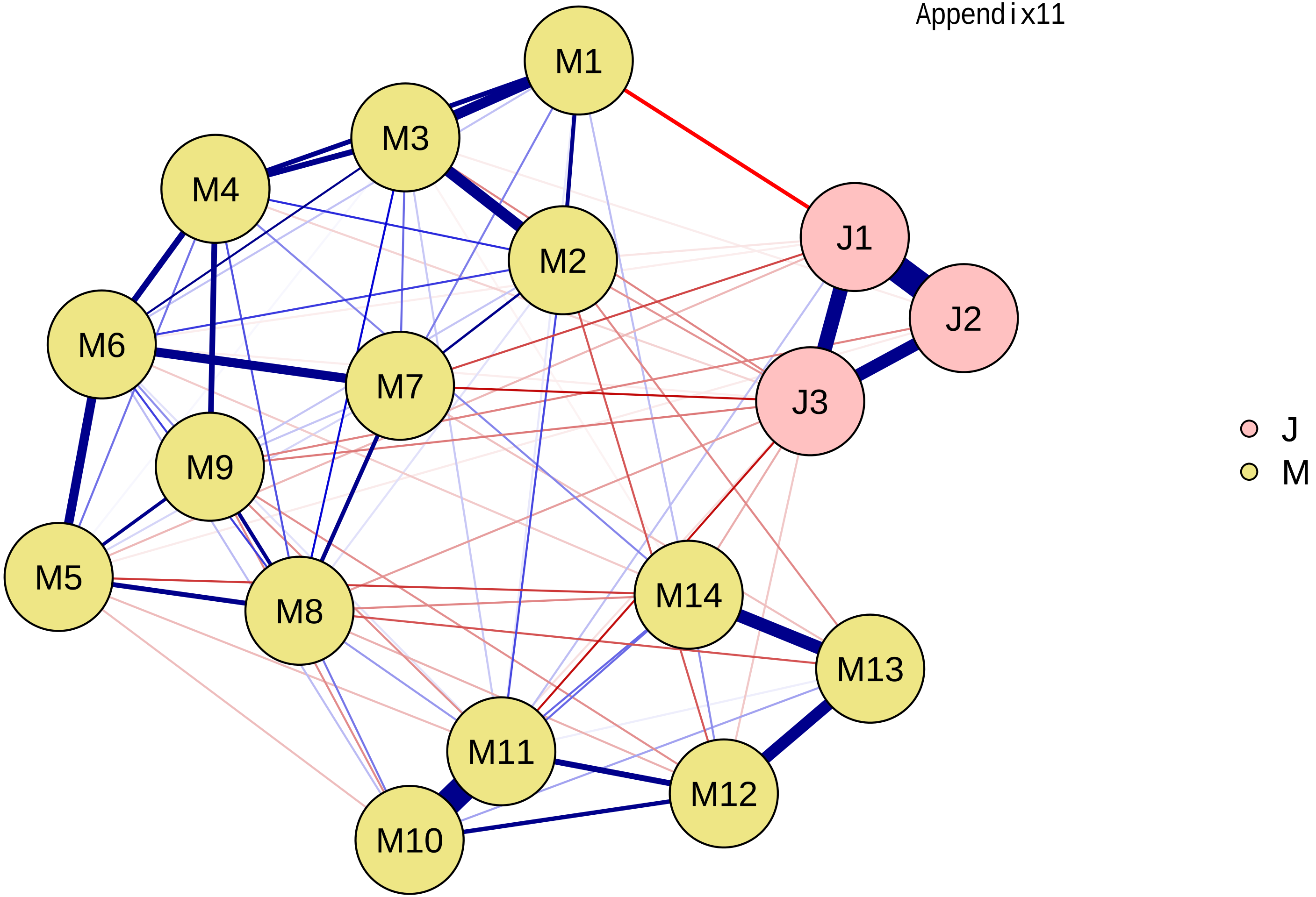

Appendix12

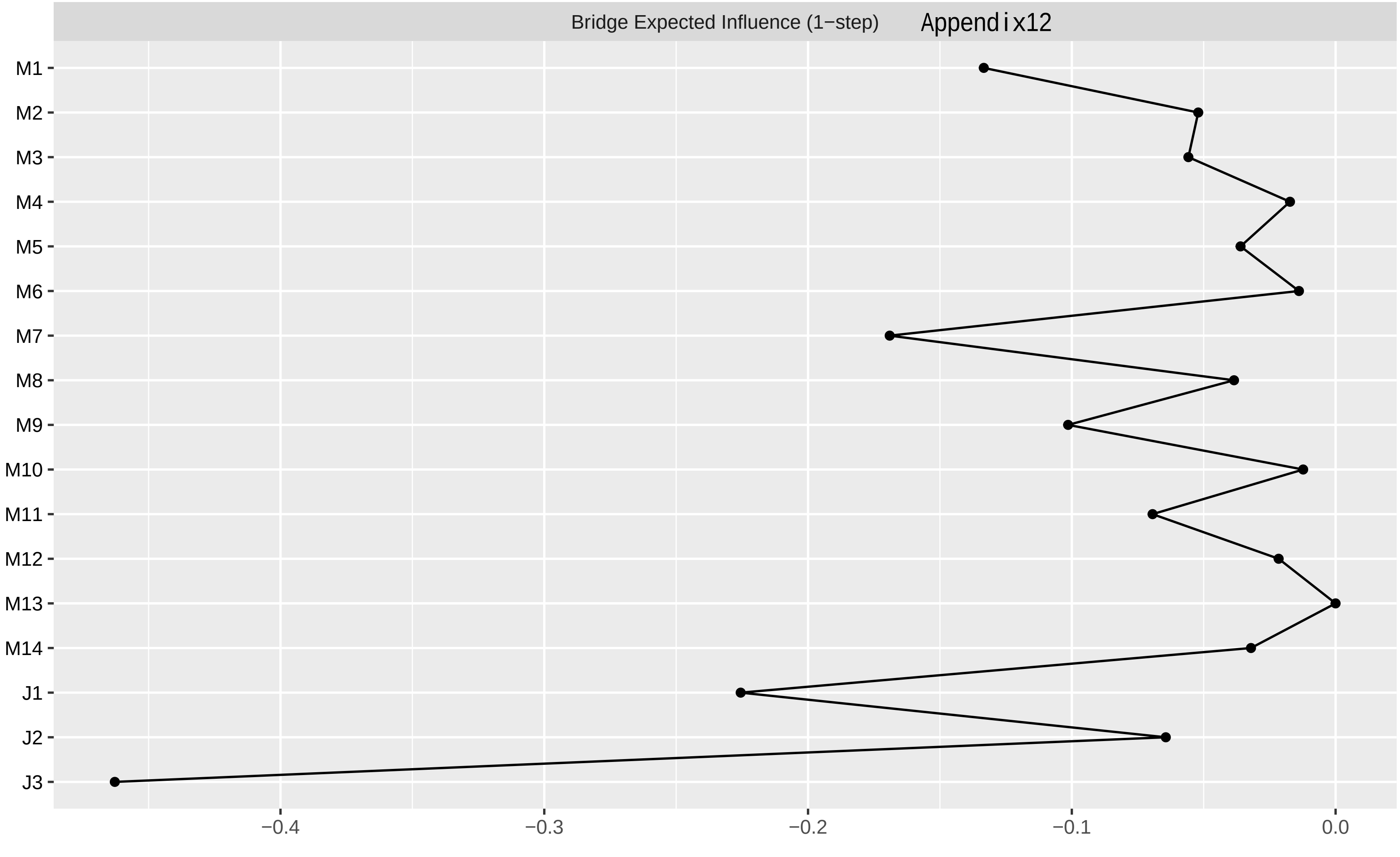

Bridge Expected Influence (1−step)
M1
M2
M3
M4
M5
M6
M7
M8
M9
M10
M11
M12
M13
M14
J1
J2
J3
−0.4
−0.3
−0.2
−0.1
0.0

Appendix13

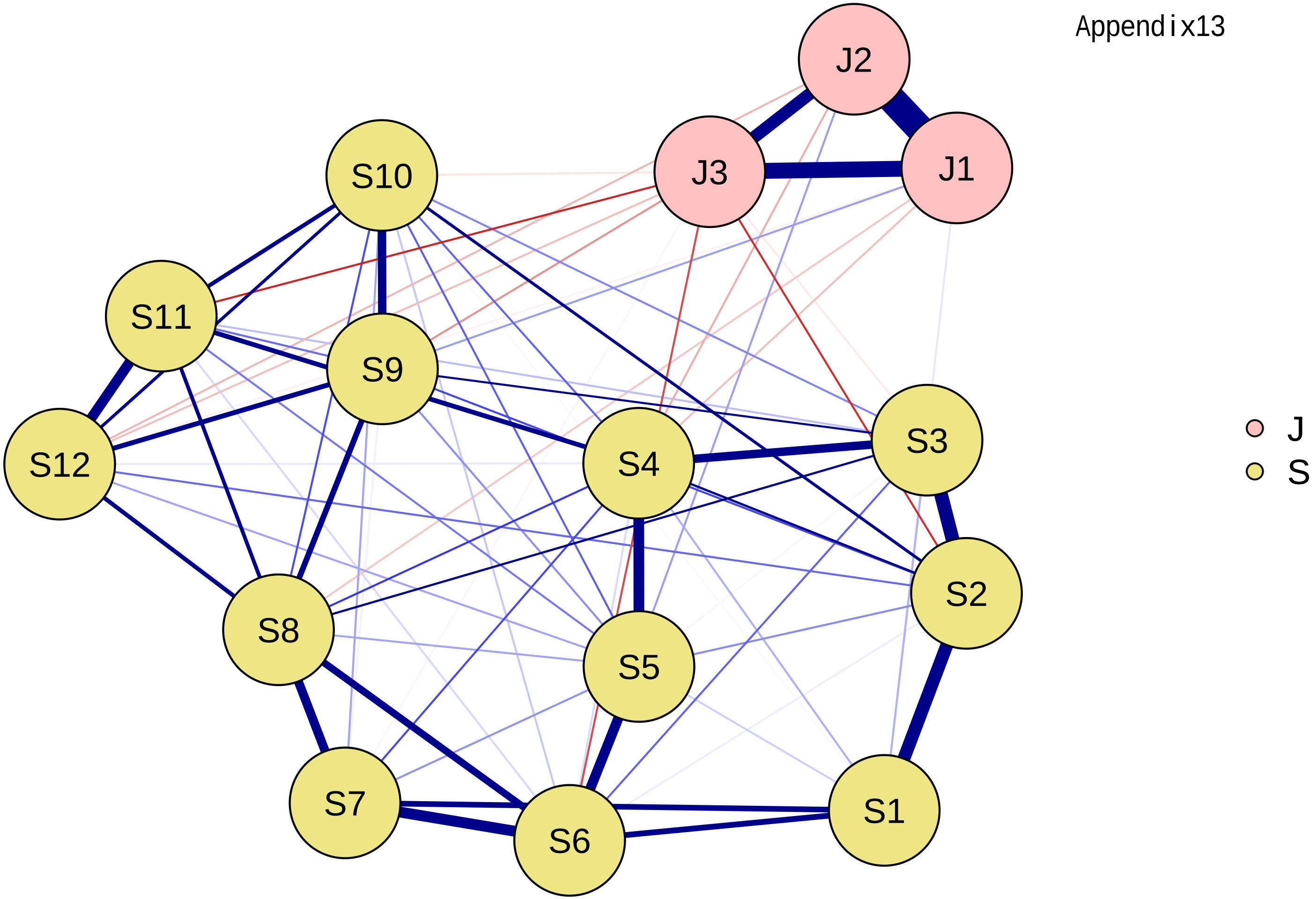

Appendix14

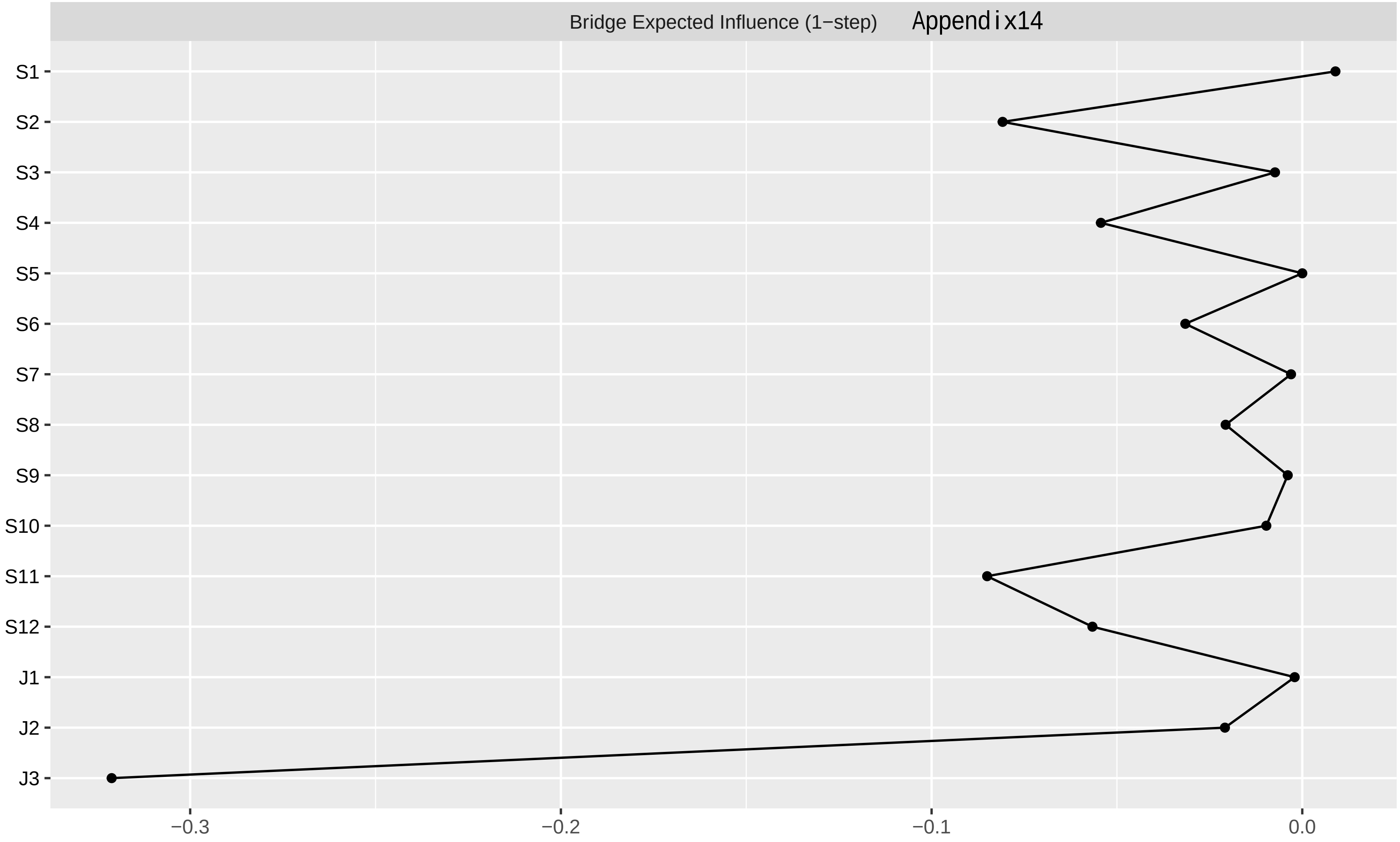
Bridge Expected Influence (1−step)
S1
S2
S3
S4
S5
S6
S7
S8
S9
S10
S11
S12
J1
J2
J3
−0.3
−0.2
−0.1
0.0
